# SOX17/PAX8 dual immunohistochemical expression in the diagnosis of Müllerian carcinomas

**DOI:** 10.1186/s13000-026-01746-2

**Published:** 2026-02-10

**Authors:** Manar Moustafa, Marwa Ahmed Mohamed Abdalrahman, Heba Mahmoud Abdelgeleel

**Affiliations:** 1 Pathology Department, Faculty of Medicine, Zagazig University, Zagazig, 11837 Egypt; 2 Obstetrics and gynecology Department, Faculty of Medicine, Zagazig University, Zagazig, Egypt

**Keywords:** SOX17, PAX8, Diagnosis, Müllerian carcinomas

## Abstract

**Background:**

Metastatic Müllerian carcinomas, including endometrial and ovarian adenocarcinomas, are challenging to diagnose due to factors like similar malignancies, insufficient clinical history, multiorgan dissemination, and small tumor specimens. Immunohistochemistry (IHC) stains are commonly used to identify these cancers. PAX8 is a widely used IHC marker with varying sensitivity levels for different types of gynecologic carcinomas. SOX17, a transcription factor involved in embryonic differentiation and development, has high specificity for ovarian and endometrial carcinomas but is weakly expressed in other epithelial neoplasms.

**Methods:**

. 56 endometrial carcinomas cases,56 ovarian cancer case and 56 cases of non-gynecological cancer, were subjected to immunohistochemical (IHC) analysis of SOX17 and PAX8.

**Results:**

In endometrial carcinomas, PAX8-high/SOX17-high co-expression strongly favored endometrioid histology (90% vs. 68.8%, *p* = 0.04), while ovarian high-grade serous carcinomas commonly expressed PAX8 (78.9% high) with heterogeneous SOX17 expression (47.4% high). Notably, double-negative PAX8/SOX17 status completely excluded Müllerian origin in metastases from colorectal, breast, and pulmonary primary tumors (100% specificity), though renal (all PAX8+) and thyroid neoplasms (63.6% PAX8+) required additional markers for distinction. Statistical analyses confirmed subtype-specific trends (*p* < 0.05 for all applicable comparisons) with loss of SOX17 associated with aggressive histotypes (25% negative in serous versus 10% in endometrioid).

**Conclusions:**

Müllerian carcinomas can be distinguished from non-gynecological metastases using PAX8 and SOX17 immunohistochemistry, with PAX8-high/SOX17-high patterns strongly indicating endometrioid differentiation and double-negative results excluding gynecologic origin with consistency in colorectal, breast, and pulmonary carcinomas. Renal and thyroid carcinomas are the diagnostic traps on the basis of PAX8 expression and require additional markers for final classification in metastatic workups.

## Introduction

The appropriate diagnosis of metastatic Müllerian carcinomas (endometrial and ovarian adenocarcinomas) remains one of the greatest challenges in surgical pathology under the following circumstances: discrimination from malignancies with similar morphology of non-gynecologic origin, insufficient clinical background history, multiorgan dissemination, or small sized tumor specimen. The precise differentiation between tumors of ovarian and endometrial origin and other sites is of very important clinical significance [1].

Metastatic cancers are frequently diagnosed using immunohistochemistry (IHC) stains. PAX8.5 is one of the most widely utilized IHC markers to identify metastatic gynecologic cancers. Various gynecologic carcinomas have various PAX8 sensitivity levels: ovarian serous carcinoma has a sensitivity of 79% to 99%, ovarian clear cell carcinoma has a sensitivity of 76% to 100%, endometrial adenocarcinoma has a sensitivity of 73% to 100%, and cervical adenocarcinoma has a sensitivity of 50%0.6. As a pan-Müllerian marker, PAX8 is also positive in some breast cancers, thyroid cancers, and renal cell carcinomas [2–7].

Conversely, SOX17 (SRY-box transcription factor 17) is involved in embryonic differentiation and development. SOX17 is found to be expressed ubiquitously within endometrial tissue as well as in many other visceral organs. SOX17 has very high specificity for ovarian and endometrial carcinomas but is weakly expressed in many other epithelial neoplasms [8–10]. According to recent research, in epithelial ovarian cancer, SOX17, a novel lineage survival transcription factor, expressed similarly to PAX8. The two genes, PAX8 and SOX17, controlled downstream genes related to tissue morphogenesis and the cell cycle [11, 12]. Furthermore, SOX17 plays a role in the development of endometrial and cervical cancer. It is believed to be a gene that suppresses endometrial adenocarcinoma tumors.

Our study investigates the diagnostic utility of SOX17/PAX8 co-expression patterns in separating Müllerian carcinomas from usual mimics. Through clinicopathological data accompanied by IHC correlation, this study aims to confirm SOX17/PAX8 double staining as a better diagnostic protocol for Müllerian carcinomas, evading pitfalls in current practices and enhancing accuracy in metastatic workups.

## Patients and methods

### Study design and case selection

Our study was divided into three parts: [1] evaluating clinicopathological and immunohistochemical expressions (SOX17-PAX8) of endometrial cancer cases; [2] evaluating clinicopathological and immunohistochemical expressions (SOX17/PAX8) of ovarian cancer cases; and [3] evaluating clinicopathological and immunohistochemical expressions (SOX17/PAX8) of non-gynecological cancer cases. The present study is based upon paraffin blocks from cases collected from the archive of Pathology Departments, Faculty of Medicine, Zagazig University during the period from January 2021 to December 2024. The clinical data of patients was collected from the report of patients at Obstetrics and gynecology Department. All the pathological sections were reviewed, and the histological diagnosis was made.

#### Inclusion Criteria

- Histologically confirmed endometrial carcinoma.
- Histologically confirmed ovarian carcinoma.
- Non-gynecologic malignancies commonly encountered in metastatic differential diagnosis: colorectal, breast, renal, thyroid, pulmonary.
- Availability of adequate paraffin-embedded tissue blocks along with complete clinical data.
- Samples collected between January 2021 and December 2024.

#### Exclusion Criteria

- These included cervical cancers, such as those associated with or independent of HPV infection, presenting as squamous or glandular tumors, since the pathogenesis and immunophenotypic features differ substantially from Müllerian endometrial and ovarian carcinomas, and since SOX17 expression is often affected by HPV-related epigenetic mechanisms. Considering cervical tumors introduces biologic heterogeneity that is not relevant to the diagnostic objective of testing SOX17/PAX8 coexpression in Müllerian carcinomas.
- Poorly preserved or too small tissue samples.
- Cases where clinical or pathological information required for comparison is not available.

### Histopathology and immunohistochemistry procedure

For each case, 3–5 μm sections were cut from formalin-fixed, paraffin-embedded (FFPE) tissue blocks. The sections were deparaffinized in two changes of xylene (10 min each), followed by rehydration through graded ethanol to distilled water. Antigen retrieval was performed by using heat-induced epitope retrieval (HIER) in citrate buffer (pH 6.0) in a microwave oven at 95–100 °C for 20 min, followed by 20 min of passive cooling at room temperature. Endogenous peroxidase activity was blocked with 3% hydrogen peroxide for 10 min.

The slides were then incubated with the following primary antibodies:

SOX17: rabbit monoclonal antibody, clone EPR20684 (Abcam, UK); working dilution 1:100, incubated for 45 min at room temperature.

PAX8: mouse monoclonal antibody, clone BC12 (Biocare Medical, USA); working dilution 1:50, incubated for 45 min at room temperature.

The Dako EnVision™ FLEX Polymer detection system (Dako, Copenhagen, Denmark) was used according to the manufacturer’s protocol. For visualization, sections were treated with 3,3’-diaminobenzidine (DAB) for 5 min followed by counterstaining with Mayer’s hematoxylin (1–2 min). Slides were dehydrated and cleared before mounting in DPX. Controls included previously validated SOX17-positive and PAX8-positive Müllerian carcinomas that were run in parallel. Negative controls were prepared by substitution of the primary antibody with non-immune serum. Only nuclear staining was considered specific for SOX17 and PAX8. The intensity/extent of staining was semiquantitatively scored as: negative (< 1%), low (1–9%), intermediate (10–49%), and high (≥ 50%).

### Statistics

Version 23 of SPSS (Statistical Package for Social Science, Chicago, Illinois, USA) was used to gather, tabulate, and statistically analyze all of the data. The frequency and percentage, which were displayed as qualitative variables, were calculated. Fisher’s exact test for categorical variables and the Mann-Whitney U test or Kruskal-Wallis test for continuous variables (Age) were used in the statistical analysis. The association between the variables was ascertained using Spearman’s correlation coefficient. Statistical significance was defined as a P-value of less than 0.05.

## Results

### The clinicopathological and immunohistochemical expressions of the 56 cases of endometrial cancer

Our study of 56 endometrial carcinomas, demonstrating significant endometrioid and serous subtype distinctions in clinicopathological variables and immunohistochemical staining patterns. The findings indicate that serous carcinomas develop in older patients (median 68 vs. 60 years, *p* = 0.02) and are exclusively high-grade (100% G3), while endometrioid tumors exhibit more variable grading. Immunohistochemically, the PAX8-high/SOX17-high co-expression pattern is far more common in endometrioid carcinomas (60% compared to 25% in serous, *p* = 0.02), consistent with diagnostic utility. SOX17 expression shows stepwise loss in the serous tumors, with 25% completely negative compared to only 10% of endometrioid, with the suggestion that SOX17 loss may be associated with high-grade histology. The extremely low numbers of double-negatives (only 1.8% overall) highlight the remarkable sensitivity of this marker combination in demonstrating Müllerian origin (Table 1).

**Table 1:** The clinicopathological and immunohistochemical expressions of the 56 cases of endometrial cancer

A- Clinicopathological features					
Characteristics		All cases *n* = 56	Endometroid *n* = 40	Serous *n* = 16	*p*-value
Age (years) Median (range)		62 (45–80)	60 (45–75)	68 (52–80)	0.02*
FIGO G1		18 (32.1%)	18 (45.0%)	0 (0%)	< 0.001*
FIGO G2		22 (39.3%)	22 (55.0%)	0 (0%)	
FIGO G3		16 (28.6%)	0 (0%)	16 (100%)	
B-Immunohistochemical expression					
PAX8	**-Ve < 1%**	2 (3.6%)	1 (2.5%)	1 (6.2%)	0.49
	**+ ve low (1–9%)**	3 (5.4%)	1 (2.5%)	2 (12.5%)	
	**+ ve intermediate (10–49%)**	8 (14.3%)	5 (12.5%)	3 (18.8%)	
	**+ ve high > 50%**	43 (76.8%)	33 (82.5%)	10 (62.5%)	
SOX17	**-Ve < 1%**	8 (14.3%)	4 (10.0%)	4 (25.0%)	0.03*
	**+ ve low (1–9%)**	5 (8.9%)	2 (5.0%)	3 (18.8%)	
	**+ ve intermediate (10–49%)**	11 (19.6%)	8 (20.0%)	3 (18.8%)	
	**+ ve high > 50%**	32 (57.1%)	26 (65.0%)	6 (37.5%)	
C- SOX17/PAX8 Dual Immunohistochemical Expression					
PAX8-high/SOX17-high		28 (50.0%)	24 (60.0%)	4 (25.0%)	0.02*
PAX8-high/SOX17-low/intermediate		15 (26.8%)	9 (22.5%)	6 (37.5%)	
PAX8-low/intermediate/SOX17-any		11 (19.6%)	7 (17.5%)	4 (25.0%)	
Double-negative (PAX8-/SOX17-)		1 (1.8%)	1 (2.5%)	0 (0%)	

*FIGO* (International Federation of Gynecology and Obstetrics)

*Fisher exact test, Mann-Whitney U test

Non-significant: P >0.05, Significant: P ≤0.05

+ve: positive, -ve :negative

### The clinicopathological and immunohistochemical expressions of the 56 cases of ovarian cancer

**Fig. 1:**
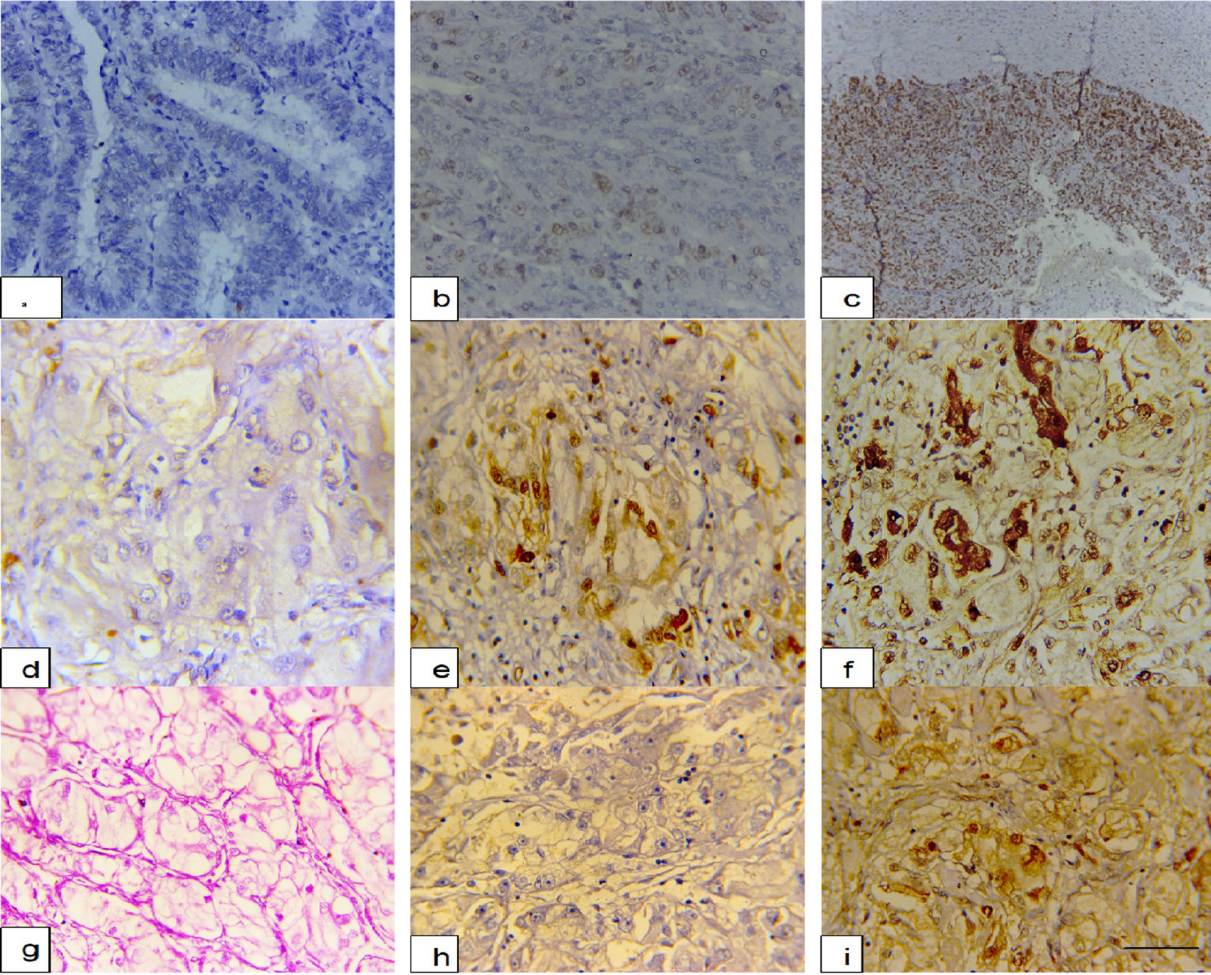
Immunohistochemical expression of SOX17 and PAX8 in endometrial carcinoma, ovarian carcinoma, and non-gynecologic tumors. **a** Endometrial carcinoma showing negative nuclear SOX17 staining. **b** Endometrial carcinoma with low-positive nuclear SOX17 staining (1–9%). **c** High-grade serous carcinoma demonstrating strong nuclear SOX17 positivity (≥ 50%). **d** Endometrial serous carcinoma with negative nuclear SOX17 staining (**e**) Endometrial carcinoma showing low-positive nuclear SOX17 expression. **f** Endometrial carcinoma with strong nuclear SOX17 positivity. **g** Renal cell carcinoma (clear cell type) showing clusters of clear malignant cells. **h** Renal cell carcinoma with negative nuclear SOX17 staining. **i** Renal cell carcinoma demonstrating strong nuclear PAX8 staining, confirming renal lineage. Scale bar = 50 μm for all images

Table 2 provides a clinicopathological and immunohistochemical correlation of the types of ovarian carcinoma, with distinct patterns of high-grade serous (HGSC), clear cell, and endometrioid carcinomas. The data reveal that HGSCs are bilateral in 78.9% and present at advanced stages (94.7% Stage III/IV). Clear cell carcinomas, while less common, show unique features such as high unilateral (60%) and early-stage (60% Stage I/II) but low SOX17 expression (30% high positivity vs. 87.5% for endometrioid). The endometrioid type shows robust PAX8/SOX17 co-expression (75% high/high), as do the endometrial endometrioid carcinomas. HGSCs are nearly universally PAX8-positive (78.9% high expression) but SOX17-variable (47.4% high), whereas endometrioid carcinomas uniformly have high SOX17 (87.5%). The highly significant p-values for the co-expression of PAX8-high/SOX17-high (*p* = 0.03) and SOX17 negativity (*p* = 0.04) highlight their diagnostic potential (Table 2; Fig. 1).

**Table 2:** The clinicopathological and immunohistochemical expressions of the 56 cases of ovarian cancer

A- Clinicopathological features						
Characteristics		All cases *n* = 56	HGSC *n* = 38	Clear cell *n* = 10	Endometroid *n* = 8	*p*-value
Age (years) Median (range)		58 (32–78)	62 (40–78)	54 (32–70)	56 (45–68)	0.08
laterality	**Unilateral**	18 (32.1%)	8 (21.1%)	6 (60.0%)	4 (50.0%)	0.02*
	**Bilateral**	38 (67.9%)	30 (78.9%)	4 (40.0%)	4 (50.0%)	
FIGO stages	I/II	12 (21.4%)	2 (5.3%)	6 (60.0%)	4 (50.0%)	< 0.001*
	**III/IV**	44 (78.6%)	36 (94.7%)	4 (40.0%)	4 (50.0%)	
B- Immunohistochemical expression						
PAX8	**-Ve < 1%**	1 (1.8%)	0 (0%)	1 (10.0%)	0 (0%)	0.15
	**+ ve low (1–9%)**	4 (7.1%)	2 (5.3%)	2 (20.0%)	0 (0%)	
	**+ ve intermediate (10–49%)**	9 (16.1%)	6 (15.8%)	2 (20.0%)	1 (12.5%)	
	**+ ve high > 50%**	42 (75.0%)	30 (78.9%)	5 (50.0%)	7 (87.5%)	
SOX17	**-Ve < 1%**	6 (10.7%)	4 (10.5%)	2 (20.0%)	0 (0%)	0.04*
	**+ ve low (1–9%)**	8 (14.3%)	6 (15.8%)	2 (20.0%)	0 (0%)	
	**+ ve intermediate (10–49%)**	14 (25.0%)	10 (26.3%)	3 (30.0%)	1 (12.5%)	
	**+ ve high > 50%**	28 (50.0%)	18 (47.4%)	3 (30.0%)	7 (87.5%)	
C- SOX17/PAX8 Dual Immunohistochemical Expression						
PAX8-high/SOX17-high		24 (42.9%)	16 (42.1%)	2 (20.0%)	6 (75.0%)	0.03*
PAX8-high/SOX17-low/intermediate		18 (32.1%)	14 (36.8%)	3 (30.0%)	1 (12.5%)	
PAX8-low/intermediate/SOX17-any		13 (23.2%)	8 (21.1%)	4 (40.0%)	1 (12.5%)	
Double-negative (PAX8-/SOX17-)		1 (1.8%)	0 (0%)	1 (10.0%)	0 (0%)	

*HGSC* (High-Grade Serous Carcinoma), *FIGO* (International Federation of Gynecology and Obstetrics)

*Fisher exact test, Kruskal-Wallis test

Non-significant: P >0.05, Significant: P ≤0.05

+ve: positive, -ve :negative

### The immunohistochemical expressions of the 56 cases of non-gynecological cancer

Table 3 demonstrates a distinct immunohistochemical discrimination between non-gynecological tumors, confirming that double-negative PAX8/SOX17 status is an ideal marker to detect colorectal, breast, and pulmonary metastases with 100% specificity, highlighting significant diagnostic pitfalls: renal tumors are always positive for PAX8 (90.9% SOX17-negative) and thyroid tumors have heterogenous PAX8 expression (63.6% positive) so other markers will be needed for accurate classification. The double-negative ratio (38/56 cases, 67.9%) illustrates that almost all Müllerian mimics can be excluded by this simple two-marker panel, but thyroid tumors are a significant exception in which 36.4% of cases may have double-negative staining (Table 3; Fig. 1).

**Table 3:** The immunohistochemical expressions of the 56 cases of non-gynecological cancer

A- Immunohistochemical expression								
Characteristics		All cases *n* = 56	Colorectal *n* = 12	Breast *n* = 11	Renal *n* = 11	Thyroid *n* = 11	Pulmonary *n* = 11	*p*-value
PAX8	**-Ve < 1%**	39 (69.6%)	12 (100%)	11 (100%)	0 (0%)	4 (36.4%)	11 (100%)	< 0.001*
	**+ ve low (1–9%)**	6 (10.7%)	0 (0%)	0 (0%)	5 (45.5%)	1 (9.1%)	0 (0%)	
	**+ ve intermediate (10–49%)**	5 (8.9%)	0 (0%)	0 (0%)	4 (36.4%)	1 (9.1%)	0 (0%)	
	**+ ve high > 50%**	6 (10.7%)	0 (0%)	0 (0%)	2 (18.2%)	5 (45.5%)	0 (0%**)**	
SOX17	**-Ve < 1%**	53 (94.6%)	12 (100%)	11 (100%)	10 (90.9%)	9 (81.8%)	11 (100%)	0.10
	**+ ve low (1–9%)**	3 (5.4%)	0 (0%)	0 (0%)	1 (9.1%)	2 (18.2%)	0 (0%)	
B- SOX17/PAX8 Dual Immunohistochemical Expression								
PAX8 +/SOX17 +		1 (1.8%)	0 (0%)	0 (0%)	1 (9.1%)	0 (0%)	0 (0%)	< 0.001*
PAX8 +/SOX17 -		17 (30.4%)	0 (0%)	0 (0%)	10 (90.9%)	7 (63.6%)	0 (0%)	
Double-negative (PAX8-/SOX17-)		38 (67.9%)	12 (100%)	11 (100%)	0 (0%)	4 (36.4%)	11 (100%)	

*Fisher exact testt

Non-significant: P >0.05, Significant: P ≤0.05

+ve: positive, -ve :negative

## Discussions

SOX17, also known as SRY-related HMG box gene 17, is a transcription factor in the SOXF subfamily. It plays a crucial role in developmental events like endoderm formation, cardiovascular and germ cell development, and hematopoiesis [13 ,14 ,15 ,16]. Recent studies suggest SOX17 may be involved in tumorigenesis, with TCGA classifying it as a mutated cancer driver gene in endometrial carcinoma. Both SOX17 and PAX8 may be involved in tumorigenesis from ovarian and endometrial origins, with PAX8 promoting angiogenesis in ovarian cancer. SOX17 may function as a tumor suppressor in many cancer types [12, 17].

Our investigation showed that the expression patterns of PAX8 and SOX17 are different. The diagnostic utility of the PAX8-high/SOX17-high co-expression phenotype was highlighted by the fact that it was significantly more common in endometrioid carcinomas (60%) than serous ones (25%, *p* = 0.02). Compared to only 10% in endometrioid cases, SOX17 expression demonstrated a significant stepwise loss in serous tumors, with 25% showing complete negativity (*p* = 0.03), indicating that SOX17 loss may be associated with higher tumor grade. Our findings, which show that SOX17 exhibits different expression patterns in endometrial carcinomas, are consistent with a number of recent studies that have found SOX17 to be a highly sensitive and specific marker for Müllerian-originated tumors. In addition to a significant number of solid tumors from other organs (*n* = 1544), Zhang et al. [1] performed immunohistochemical staining on a large cohort of ovarian and endometrial cancers (*n* = 416). They discovered that SOX17 was highly expressed in various subtypes of ovarian carcinoma (97.5% in serous, 90% in endometrioid, and 100% in clear cell carcinomas) and endometrial carcinomas (88% in endometrioid, 100% in serous and clear cell carcinomas). Also, these results were supported by another thorough investigation, which found that SOX17 is a highly specific marker for Müllerian origin in about 94% of endometrial tumors and absent in almost all other tumor types [18].

Our study found that serous carcinomas had lower SOX17 expression (25% completely negative) than endometrioid tumors (10% completely negative), which might indicate underlying molecular differences between the two subtypes. The rate of SOX17 mutations in endometrioid endometrial carcinomas was 11.5%, with frameshift mutations accounting for over half of the mutations ‎[19]. Also, this study found that low/absent SOX17 staining was significantly associated with advanced stage, high tumor grade, and reduced recurrence-free survival [19], which is consistent with our findings of more frequent SOX17 loss in high-grade serous carcinomas. However, it did not find a direct correlation between mutation status and protein expression.

Our findings, which demonstrate that PAX8 expression is present in 96.4% of endometrial carcinomas overall, are consistent with previous research on this well-established marker of Müllerian origin. The finding that high-level PAX8 expression (> 50% of tumor cells) was present in 82.5% of endometrioid and 62.5% of serous carcinomas is in line with a thorough investigation by Yemelyanova et al. that assessed PAX8 expression in a variety of uterine adenocarcinomas and discovered positive staining in 95% of serous carcinomas and 96% of endometrioid carcinomas ‎[20]. Their research did point out, though, that serous carcinomas showed a noticeably greater degree of expression (based on combined extent and intensity) than endometrioid carcinomas—a subtlety that our semi-quantitative evaluation might not have adequately captured.

Our results should be interpreted against the potential influence of FIGO stage on SOX17 and PAX8 expression. As a lineage-survival transcription factor, PAX8 expression has been reported in previous studies to be unchanged across the progression of disease and not affected by tumor stage. Similarly, the down-regulation of SOX17 has been associated with more aggressive biological behavior, including promoter methylation in high-grade or advanced-stage endometrial carcinoma. No statistically significant correlation was identified in our cohort between FIGO stage and marker expression across either the ovarian or endometrial subgroups, likely reflecting their characteristic biology (e.g., high-grade serous carcinomas frequently present at advanced stages yet retain high PAX8 expression). Nonetheless, we recognize the potential for heterogeneity within stage-dependent biology to impact SOX17 expression, and larger studies that are balanced for stage are required to further elucidate this relationship.

Our study’s most important discovery is the diagnostic value of combining SOX17 and PAX8 immunohistochemistry; endometrioid carcinomas have a significantly higher frequency of the PAX8-high/SOX17-high pattern (60% vs. 25% in serous, *p* = 0.02). With significant ramifications for clinical management and prognosis prediction, this differential expression pattern offers a potentially useful tool for differentiating between these histological subtypes. The remarkable sensitivity of this marker combination in confirming Müllerian origin is highlighted by the incredibly low rate of double-negative cases (1.8% overall).

Our findings strongly support and validate the traditional PAX8 role as a lineage-restricted marker. PAX8 negativity in all colorectal, breast, and pulmonary carcinomas in our series is consistent with large studies that have definitively shown these carcinoma types are typically PAX8-negative [21]. This pattern is the foundation for the 100% specificity for the PAX8-/SOX17- phenotype for rejecting Müllerian origin for these metastases. Furthermore, our results in renal tumors, which were all PAX8-positive (100%), are consistent with literature reporting PAX8 expression in approximately 90% of renal cell carcinomas. The heterogeneous PAX8 expression found in our thyroid tumors is also a characteristic aspect, as PAX8 is a well-documented regulator of thyroid organogenesis and a sensitive marker for thyroid carcinomas, albeit its expression can be heterogeneous [21, 22].

The key finding from our diagnosis is the established utility in pairing SOX17 with PAX8. While PAX8 is highly sensitive for Müllerian, renal, and thyroid malignancies, it is not specific to differentiate among these lineages. Our data indicate that SOX17, a highly sensitive and specific immunomarker for endometrial and ovarian carcinomas, can solve this dilemma. For instance, the finding of 90.9% of renal tumors within our series to be SOX17-negative creates a PAX8+/SOX17- profile strongly typical of a renal primary. This is corroborated by recent literature on mesonephric-like adenocarcinoma (MLA), which likewise found that though combination of strong expression of PAX8 with loss or focal weak expression of SOX17 may prove useful in diagnosing this extremely rare tumor type [23]. Therefore, the SOX17-/PAX8 + signature effectively distinguishes renal and several MLA tumors from most of Müllerian carcinomas that share the expression of both markers.

Accumulating evidence shows that PAX8 and SOX17 are not simply diagnostic lineage markers but active molecular contributors to the pathogenesis of Müllerian carcinomas. PAX8 is a lineage-survival transcription factor responsible for maintaining the identity of fallopian tube secretory epithelium and promotes tumorigenesis by controlling genes in cell proliferation, survival, and angiogenesis[‎11]. In contrast, SOX17 is a context-dependent regulator-that is, it acts as a tumor suppressor in endometrial carcinoma, where its loss reduces differentiation and increases aggressiveness in tumors, whereas in ovarian carcinoma, it cooperates with PAX8 in a transcription complex to control pathways crucial in cell-cycle progression and tissue morphogenesis. Thus, the co-expression patterns in our cohort likely reflect these underlying biological processes: PAX8-high/SOX17-high expression in endometrioid tumors reflects preserved Müllerian differentiation, while stepwise SOX17 loss in serous carcinomas recapitulates high-grade behavior and progression. These mechanisms help explain the subtype-specific expression patterns identified in our study.

Finally, the most relevant diagnostic dilemma highlighted by our results as well as the literature is the proper identification of thyroid tumors. Our results show that 36.4% of thyroid cases presented a double-negative (PAX8-/SOX17-) profile, which resembles that of colorectal, breast, and pulmonary metastases and represents a major diagnostic pitfall. This serves to emphasize the need, as we stated, for additional markers in challenging cases for diagnosis. To accurately classify a PAX8-/SOX17- tumor or a PAX8 + tumor of indeterminate lineage, such as lineage-specific markers such as TTF-1 for lung and thyroid, GATA3 for breast and select renal neoplasms, or CDX2 for colorectal origin is imperative.

In conclusion, our study confirms the utility of a two-marker panel of SOX17 and PAX8 as a valuable first-line triage tool for carcinomas of unknown primary, but that it must be used with acknowledgment of its limitations, particularly in the context of thyroid neoplasia, and supported by an expanded immunohistochemical panel to render a definitive diagnosis.

### Limitation

The relatively low sample number, especially in the serous carcinoma subgroup, is a limitation of our retrospective design. This reflects, however, the natural case distribution within our institution and also the necessity of sufficient paraffin-embedded material and complete clinical data. Besides, serous carcinoma is a well-accepted high-grade malignancy and, under current WHO and FIGO criteria, is routinely classified as FIGO grade 3; thus, the uniform G3 status of our cases reflects the intrinsic biology of this subtype. Having said this, our analysis still resulted in statistically significant differences in the expression of PAX8 and SOX17 across tumor types. Future multi-center studies with larger cohorts are needed to validate the generalizability of our findings.

## Data Availability

No datasets were generated or analysed during the current study.
